# Mifepristone and Misoprostol vs Misoprostol Alone in Second Trimester Termination of Pregnancy

**DOI:** 10.31729/jnma.3690

**Published:** 2018-10-31

**Authors:** Deepa Shah, Pappu Rijal, Achala Thakur, Rubina Rai

**Affiliations:** 1 Department of Obstetrics and Gynaecology, B.P. Koirala Institute of Health Sciences, Dharan, Nepal

**Keywords:** *abortion*, *mifepristone*, *misoprostol*, *second trimester termination*

## Abstract

**Introduction:**

During the last decade, medical methods for second trimester abortion have considerably improved and become safe and more accessible. The combination of mifepristone and misoprostol is now an established and highly effective method for second trimester abortion. But where mifepristone is not available or affordable, misoprostol alone has also been shown to be effective. The objective of this study is to compare the efficacy of mifepristone with misoprostol and misoprostol alone for second trimester termination of pregnancy.

**Methods:**

It is a comparative study conducted on 60 patients from 13 to 18 weeks of gestation admitted for second trimester termination on legal indications.

**Results:**

Mean induction abortion interval was comparable in both the groups. Of the 30 cases in each group, nine cases in each Group A and six cases in Group B had incomplete/failed expulsion. Among these 15 cases, only nine required check curettage for complete evacuation while others received oxytocics only for completion. The distribution of these cases was also comparable in both the groups. Only one patient in Group B had complete failure of expulsion and underwent surgical evacuation. However, the difference in dosage of misoprostol required for complete expulsion and incidence of side effects were significantly higher in the group B.

**Conclusions:**

Mifepristone and misoprostol combined together is now an established, highly effective and safe method for medical method of second trimester termination. However, when mifepristone is not available or affordable, misoprostol alone can also be used effectively, although a higher total dose is needed and side effects are higher than with the combined regimen.

## INTRODUCTION

Second trimester termination of pregnancy is a social, emotional and management challenge that most clinicians would be glad to avoid. Second trimester abortions constitute 10–15% of all induced abortions worldwide but are responsible for two-thirds of major abortion-related complications.^1^

The combination of mifepristone and misoprostol is now an established and highly effective method for second trimester abortion. Where mifepristone is not available or affordable, misoprostol alone has also been shown to be effective, although a higher total dose is needed and efficacy is lower than for the combined regimen.^1^ If the efficacy of misoprostol alone is found to be comparable to misoprostol combined with mifepristone, second trimester termination will be a cheaper procedure and will require shorter hospital stay as mifepristone is a much expensive drug (NPR 600 per tablet compared to NPR 20 per tablet) and needs to be administered 24 hours prior to misoprostol. The aim of this study was to compare both the regimens and their efficacy.

## METHODS

A comparative study was conducted to compare the efficacy of misoprostol alone with misoprostol and mifepristone in the Department of Obstetrics and Gynaecology, BPKIHS, Dharan, from August 2015 to July 2016. The study population was cases admitted to the inpatient department for termination of second trimester pregnancy on legal indications from 13 to 18 weeks of gestation. Patients with known contraindication to the use of mifepristone or misoprostol, prolonged corticosteroid therapy, chronic adrenal failure, inherited porphyrias or hypersensitivity reaction to mifepristone or misoprostol were excluded from the study.

The sample population was randomly divided into 2 groups using computer-generated random number table. Group A received tablet mifepristone 200 milligrams (mg) per oral stat followed by tablet misoprostol 400 micrograms (mcg) per vaginum four hourly till establishment of active labor or up-to five doses. Group B received tablet misoprostol 400 mcg per vaginum four hourly till the establishment of active labor or up-to five doses. In case of failure in either group, the patient was given rest for 24 hours and received the next cycle of tablet misoprostol thereafter. In case of failure again, the patient underwent surgical management (dilatation and evacuation).

**Figure 1.**
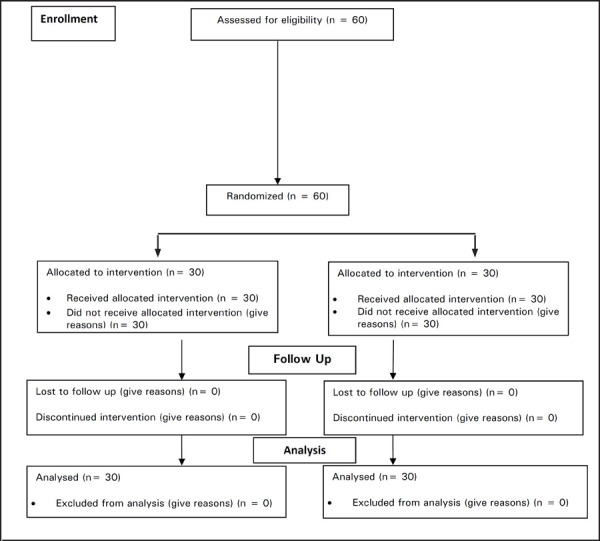
Consort Flow diagram of the study.

This study considered 95% confidence interval and 90% power for sample size estimation.

Based on the literature,^2^ considering the mean induction abortion interval, the sample size was calculated using the formula given below.


$$\begin{array}{l} n=\frac{2\left(\bar{p}\right)\left(1-\bar{p}\right){\left(Z_{\beta }+Z_{\alpha /2}\right)}^{2}}{{\left({\text{p}}_{1}-p_{2}\right)}^{2}}\\ \text{n}=14\text{in each group} \end{array}$$

n=14 in each group

Adding 10% in calculated sample size to reduce various types of biases, sample was calculated to be 16 in each group. But because the large sample theory of statistics recommends >30 samples, this study considered 30 sample in each arm (Total 60).

The ethical clearance was received from Institutional Ethical Review Board of B.P. Koirala Institute of Health Sciences, Dharan. Informed written consent was obtained from each participant. Data were entered in Microsoft Excel 2007 and converted into SPSS 11.5 version for statistical analysis. Data were analyzed by using percentage, proportion, mean, SD, median, interquartile range to describe it. Chi-square test, independent t-test or Mann Whitney U test were applied to find out the significant differences between the two groups (at 95% CI where the level of significance is 0.05).

## RESULTS

All the baseline characteristics and obstetric profile of cases in both the groups were comparable as shown (Table 1).

**Table 1.** Baseline characteristics.

Characteristics	Category	Group Mifepristone + Misoprostol	Misoprostol alone	P
**Age (in years)**	Mean age±SD	24.90±5.42	27.27±7.27	0.158
**Marital status**	Married	25 (48.1%)	27 (51.9%)	
	Single	5 (62.5%)	3 (37.5%)	0.706
**Parity**	Median (IQR)	2 (1.75–3)	2.5 (1 - 4)	0.538
	Period of gestation by LMP	15.27±1.46	15.47±1.78	0.636
**Period of gestation (in weeks)**	Period of gestation by USG	14.97±1.60	15.07±1.74	0.818
	Uterine size	15.40±1.75	15.47±1.73	0.883
**MA/ MVA failure**	Yes	8 (40.0%)	12 (60.0%)	0.273
	No	22 (55.0%)	18 (45.0%)	

**Figure 2.**
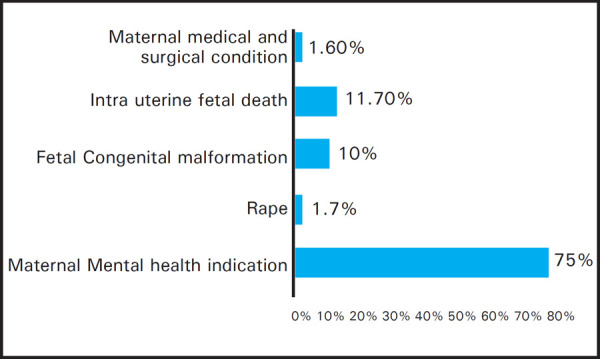
Indication for termination of pregnancy.

Majority of cases were terminated for maternal mental health indications and the distribution of cases was similar in both the groups. Maternal mental health indication included cases with contraception failure and failed medical abortion/ manual vacuum aspiration (Figure 2).

The analyzed data of both the groups were compared in terms of the mean induction-abortion interval, completeness of the expulsion of the fetus along with placenta, the dosage of misoprostol required for complete expulsion in both groups, need of additional intervention and side effects of drugs.

The induction-abortion interval was found to be higher in misoprostol alone group but the difference was not statistically significant (Table 2). Of the total cases, 45 (75%) cases had complete expulsion and remaining had incomplete/failed expulsion and required other means of evacuation. The distribution of these cases was also comparable in both the groups. Only one patient (1.67%) had complete failure of expulsion after two cycles of misoprostol and underwent surgical evacuation and it belonged to the group receiving misoprostol only.

Amongst the 15 cases with incomplete/failed expulsion, only nine patients required additional methods of evacuation i.e. check curettage for completion of expulsion. The remaining received other oxytocics like oxytocin and had spontaneous expulsion thereafter and did not require any additional intervention. Again, the cases with failed/incomplete expulsion were comparable in both the groups.

**Table 2:** Outcomes of the study.

Outcomes		Mifepristone and misoprostol	Misoprostol alone	P
Induction-abortion interval (in hours) Median (IQR)		12.32 (8.78–15.06)	14.75 (9.43–18.25)	0.056
Dosage of misoprostol required for complete expulsion Mean±SD		2.83±0.99	3.63±1.62	0.025
Failed/ incomplete	Yes	9 (60.0%)	6 (40.0%)	0.371
expulsion	No	21 (46.7%)	24 (53.3%)	
Additional methods of evacuation	Yes	4 (44.4%)	5 (55.6%)	1.0
	No	26 (51.0%)	25 (49.0%)	
Side effects	Yes	10 (33.3%)	20 (66.7%)	0.010
	No	20 (66.7%)	10 (33.3%)	

## DISCUSSION

Second trimester medical abortion regimens have evolved greatly over the past 20 years with increasing availability of prostaglandin analogs and anti-progesterone agents such as mifepristone. Older regimens such as instillation of hypertonic saline or prostaglandin F2a although effective in provoking abortion were associated with higher rates of serious adverse events that are modern methods.^3^

Medical abortion (MA) involves the use of misoprostol and mifepristone. Combination of mifepristone and misoprostol has become the most adopted regimes nowadays. Misoprostol, a newer synthetic prostaglandin E1, has proven its efficacy as an abortifacient for second trimester termination since 1987.^4^ Aim of this study was to investigate misoprostol alone or in combination with mifepristone is effective in second trimester abortion.

In this study, the median induction-to-abortion interval in the group receiving both mifepristone and misoprostol was 12.32 hours whereas that in the group receiving misoprostol only was 14.75 hours. A similar study was conducted by Patel U et al^5^ in Piparia, India amongst 50 cases, 25 in each group, using tablet misoprostol 200 mcg 6 hours apart and the induction abortion interval was found to be 18.94 hours among the women in the first group and 24.29 hours in the women who were in the second group, the difference being statistically significant.

Although the difference in the interval was not found to be statistically significant in this study, the shorter induction-abortion interval in the group receiving mifepristone combined with misoprostol could be because of the anti-progesterone action of mifepristone blocking the progesterone receptors and sensitizing the uterus to the activity of the prostaglandins, thus, improving the efficacy of the misoprostol in the process of termination. This difference in the results between the two studies could be due to the difference in the dosage of misoprostol used and the difference in the dosing interval between two dosages. Lower dose of misoprostol and greater interval between two dosages could be a reason for longer induction abortion interval in the later study.

The results of this study brought out that 60% of the incomplete abortion occurred in the group receiving mifepristone, but the difference was not statistically significant. Similarly, Nagaria et al^2^ also concluded that the cases receiving mifepristone also required additional methods of evacuation less frequently but the difference was not statistically significant. In contrast, Patel U et al^5^ stated that no case who received mifepristone had incomplete expulsion and 4 cases who did not receive mifepristone expelled incompletely.

Also, this study found that the side effects were significantly lower in the cases receiving mifepristone than their counterparts not receiving it. A similar difference in side effects was noted in the studies by Nagaria et al^2^ and Patel U et al.^5^ All the studies concluded that there was statistically significant difference in the occurrence of side effects in both the groups. This difference could be because of the higher dosage of misoprostol required for expulsion of the products of conception.

With this, it can be safely articulated that pre-treatment with tablet mifepristone 200 mg provides an effective, non-invasive medical second trimester termination and significantly reduces the dosage of misoprostol and its side effects.

As this study was carried out in a small scale with a small sample size, further studies with larger sample size are required to draw definitive conclusion on the efficacy of misoprostol with or without mifepristone. Therefore, whenever possible, the combined regimen should be used until further studies are available to support the efficacy of misoprostol without the use of mifepristone. But both the regimens are feasible as far as end results are concerned and misoprostol-only regimen can be used when mifepristone is not available or affordable.

## CONCLUSIONS

The combination of mifepristone and misoprostol is now an established and highly effective and safe method for medical method of second trimester abortion. The combination of misoprostol reduces the induction-abortion interval, dosage of misoprostol required for complete expulsion and also has fewer side effects. But at instances, where mifepristone is not available or affordable, misoprostol alone has also been shown to be effective, although a higher total dose is needed and has side effects which are higher than with the combined regimen.

## Conflict of Interest

**None.**
